# "Pardon My Language": The Curious Case of a Bloody Sandwich and the John Cunningham Virus Reactivation in the Era of Immunomodulatory Drugs

**DOI:** 10.7759/cureus.72306

**Published:** 2024-10-24

**Authors:** Francis Guerra-Bauman, Jessica E Tseng, Asfandyar Latif, Chloe Cohan, Jason Assalita, Abdul Waheed

**Affiliations:** 1 Department of Family Medicine, Good Samaritan Hospital, Lebanon, USA; 2 Department of Family Medicine, Creighton University School of Medicine, Phoenix, USA; 3 Department of Family Medicine, WellSpan Good Samaritan Hospital, Lebanon, USA; 4 Department of Family Medicine, Family Medicine Residency Program, WellSpan Good Samaritan Hospital, Lebanon, USA; 5 Department of Family and Community Medicine, Creighton University School of Medicine, Phoenix, USA; 6 Department of Family Medicine, Dignity Health Medical Group, Gilbert, USA

**Keywords:** immunosupressants, jc virus, multiple myeloma, pomalidomide, progressive multifocal leukoencephalopathy

## Abstract

Progressive multifocal leukoencephalopathy (PML) is a rare diagnosis associated with high mortality in different clinical settings. PML has been attributed to the reactivation of the John Cunningham (JC) virus (JCV). JCV typically affects patients with HIV/AIDS, solid organ and hematological malignancies, and those under treatment with immunomodulatory drugs (IMiDs) like pomalidomide. Currently, there are a limited number of reported cases of patients with multiple myeloma (MM) who developed PML reported in the literature, and one case report of a patient with MM diagnosed with PML in association with pomalidomide and daratumumab. Here, we describe a case of PML in a patient with MM status post autologous stem cell transplant (ASCT) on maintenance treatment with pomalidomide who presented with cognitive decline and aphasia. In an era of increased access to immunomodulatory therapies, physicians should be able to recognize this potential complication as discontinuation of the medication could be lifesaving.

## Introduction

Progressive multifocal leukoencephalopathy (PML) is a rare demyelinating disease of mainly the central nervous system associated with high mortality in medical practice [1]. PML has been reported as a complication of reactivation with the John Cunningham virus (JCV) and largely affects patients with HIV/AIDS, solid organ and hematological malignancies, and those under treatment with immunomodulatory drugs (IMiDs) like thalidomide, lenalidomide, and pomalidomide. In patients with hematologic malignancies such as multiple myeloma (MM), prolonged immunosuppression from IMiDs can increase the risk of JCV reactivation, leading to PML. JCV infection is ubiquitous, typically transmitting through direct contact, traveling through the oropharynx, and spreading hematogenously into sites such as the kidney, bone marrow, and lymphoid tissue. Prolonged impairment of cellular immunity allows JCV to reactivate and travel via leukocytes to the CNS, causing multifocal demyelination swelling and death of oligodendrocytes and astrocytes [2].

While JCV reactivation is typically asymptomatic, early signs of JCV reactivation may be detected through laboratory tests, such as the presence of the virus in cerebrospinal fluid (CSF) or an increase in JCV DNA levels in the blood [3,4]. Regular monitoring in high-risk patients, such as those on immunosuppressive therapies or IMiDs, can help in early detection and management and thus better outcomes. PML, though rare, should be considered in the differential diagnosis for all patients presenting with neurocognitive abnormalities, such as cognitive impairment, aphasia, ataxia, hemianopia, or hemiparesis, particularly in those who are immunocompromised [5].

IMiDs are commonly used in the treatment of patients with MM due to their immunomodulatory and anti-cancer properties. MM is a hematologic malignancy characterized by aberrant proliferation of clonal bone marrow plasma cells, hypercalcemia, renal insufficiency, anemia, and osteolytic bone lesions. Significant advancements in pharmacotherapy to reduce malignant plasma cells have increased patient survival rates. In the US, first-line treatment for newly diagnosed MM is a three-drug regimen consisting of lenalidomide, bortezomib, and dexamethasone. Eligible patients may be considered for autologous hematopoietic stem cell transplantation [6]. Due to this new standard of implementing IMiDs in the treatment of MM, patients are severely immunosuppressed for an extended period.

A systematic search using Medical Subject Headings (MeSH) terms “Multiple Myeloma” AND “Progressive Multifocal Leukoencephalopathy” AND “Immunomodulatory Drugs” was conducted in PubMed (National Library of Medicine) on August 15, 2024, and revealed only 17 cases. Some previous case reports suggest that preceding therapy with IMiDs is the causative agent behind PML in patients with MM, rather than MM itself. Among these cases, preceding IMiDs include five accounts of lenalidomide, two of thalidomide, and three of pomalidomide. However, given the limited number of cases and confounding variables, such as T-cell dysregulation in MM or concurrent use of steroids and chemotherapy drugs, a strong causative link between IMiDs and PML remains difficult to establish [7,8].

We present a case of PML in a patient with MM status post-autologous stem cell transplant (ASCT) on maintenance treatment with pomalidomide who presented with cognitive decline and aphasia and was found to have JCV infection as well.

## Case presentation

This is a case of a 66-year-old male with a medical history of MM, status post-ASCT two years ago, currently on pomalidomide. The patient was diagnosed with MM three years before presentation after a bone biopsy showed CD 128-positive diffuse plasma cell myeloma. Initially, he was started on lenalidomide at a dose of 15 mg for 21 days out of a 28-day cycle, which was later increased to 25 mg daily. He was also treated with carfilzomib 110 mg every 28 days, daratumumab 740 mg every 28 days, and dexamethasone (decadron) 20 mg weekly. The patient underwent a melphalan-based ASCT two years before presentation and was shortly afterward started on pomalidomide 4 mg daily for 21 days out of a 28-day cycle. His past medical history also included non-insulin-dependent type 2 diabetes, essential hypertension, hyperlipidemia, and obstructive sleep apnea.

He presented to the emergency department accompanied by his family due to a recent acute decline in mental status, characterized by worsening encephalopathy, difficulty with word finding, and unusual behavioral requests, such as asking for a *blood sandwich*. The family had observed these behavioral changes over the preceding months, with a noticeable deterioration in the past few days before the presentation. According to the family, the patient appeared to lack insight into his condition. On physical exam, the patient was noted to be alert and oriented to self and place, but not to time. The neurological exam did not reveal any cranial nerve deficits, motor impairments, or sensory impairments. The National Institutes of Health Stroke Scale (NIHSS) score was recorded as 3, indicating aphasia and disorientation over time. Additionally, the patient exhibited repetitive involuntary movements of the oral, lingual, and buccal areas (lip-smacking). Routine laboratory investigations were noted to be normal along with a negative urine toxicology (Table 1).

**Table 1:** Initial laboratory findings upon presentation to the emergency department. WBC, white blood cell; RBC, red blood cell; MCV, mean corpuscular volume; MCH, mean corpuscular hemoglobin; MCHC, MCH concentration; RDW-SD, red cell distribution width with standard deviation; RDW-CV, red cell distribution width with coefficient of variance; HDL, high-density lipoprotein; nRBC, nucleated red blood cell; CO_2_ is carbon dioxide; BUN, blood urea nitrogen; AST, aspartate transferase; ALT, alanine transferase; HIV, human immunodeficiency virus

Laboratory parameter	Patient lab values	Normal range
WBC	2.0 K/mcL	4.0-11.0 K/mcL
RBC	3.95 M/mcL	4.30-5.90 M/mcL
Hemoglobin	13.3 g/dL	13.0-17.3 g/dL
Hematocrit	39.1%	38.5%-53.0%
MCV	99.0 fL	83.0-99.0 fL
MCH	33.7 pg	27.0-33.0 pg
MCHC	34.0 g/dL	31.0-35.0 g/dL
Platelets	137 K/mcL	140-400 K/mcL
Mean platelet volume (MPV)	9.7 fL	9.0-12.6 fL
RDW-SD	55.8 fL	37.0-53.1 fL
RDW-CV	15.3%	11.0%-16.5%
nRBC %	0.0%	0.0%-0.0%
Absolute nRBC by automated count	0.00 K/mcL	<1.00 K/mcL
Triglycerides	244 mg/dL	0-150 mg/dL
Total cholesterol	194 mg/dL	0-200 mg/dL
HDL cholesterol	34 mg/dL	>40 mg/dL
Calculated LDL cholesterol	111 mg/dL	<100 mg/dL
Calculated non-HDL cholesterol	160 mg/dL	<130 mg/dL
Glucose	137 mg/dL	70-139 mg/dL
Sodium	143 mmol/L	135-145 mmol/L
Potassium	3.3 mmol/L	3.5-5.3 mmol/L
Chloride	108 mmol/L	98-107 mmol/L
CO_2_	26 mmol/L	21-31 mmol/L
Anion gap	9 mmol/L	3-11 mmol/L
BUN	15 mg/dL	7-25 mg/dL
Creatinine	1.15 mg/dL	0.70-1.30 mg/dL
Calcium	9.2 mg/dL	8.6-10.3 mg/dL
Total protein	6.5 gm/dL	6.4-8.9 gm/dL
Albumin	4.3 gm/dL	3.5-5.7 gm/dL
Alkaline phosphatase	32 IU/L	34-104 IU/L
AST	10 IU/L	13-39 IU/L
ALT (SGPT)	13 IU/L	7-52 IU/L
Total bilirubin	0.4 mg/dL	0.3-1.0 mg/dL
Hemoglobin A1C	6.9%	<5.7%
Thiamine	8 nmol/L	8-30 nmol/L
Vitamin B12	810 pg/mL	180-914 pg/mL
Ammonia	23 mcmol/L	17-60 mcmol/L
HIV antigen/antibody	Nonreactive	Nonreactive

**Figure 1:**
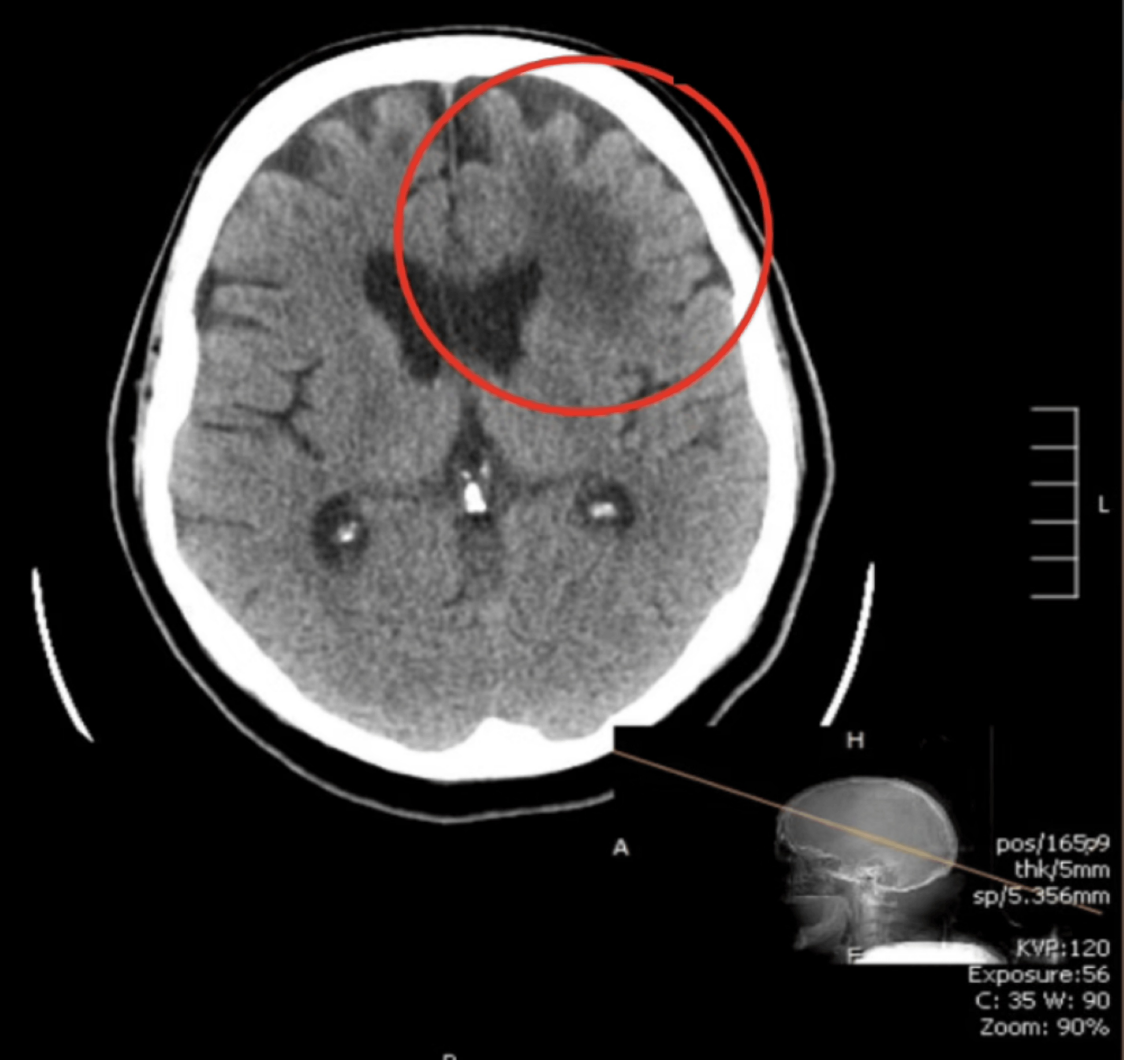
CT scan of the head showing an area of hypodensity in the left frontal lobe, marked with a circle.

**Figure 2:**
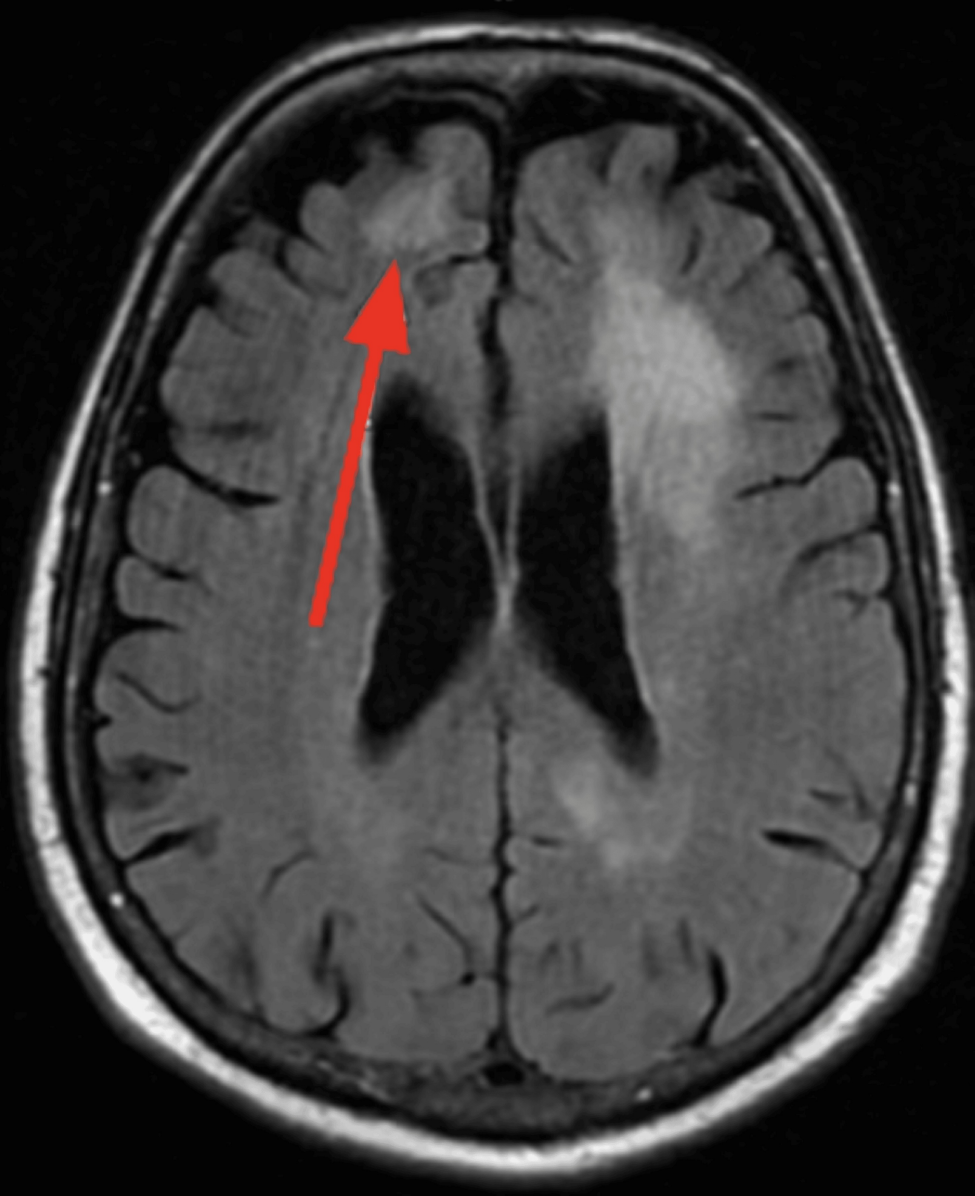
MRI of the brain showing an area of hypodensity in the left frontal lobe, marked by the arrow.

Computed tomography (CT) of the head without contrast showed an area of hypodensity in the left frontal lobe (Figure 1). Magnetic resonance imaging (MRI) head showed multiple periventricular subcortical areas of high signal abnormalities, with no suggestion of acute infarct; however, late subacute infarcts were noted; sequelae of infectious inflammatory disease cannot be completely excluded (Figure 2). 

A lumbar puncture was performed, revealing normal glucose levels, slightly elevated protein levels, and no bacterial or fungal growth. Pathological examination showed no evidence of malignant cells. However, the JCV polymerase chain reaction (PCR) was noted to be positive. At this point, the patient was diagnosed with PML.

The patient's pomalidomide treatment was discontinued. Both the patient and their family were provided with education and support. Subsequent evaluations by hematology and neurology led to a recommendation for palliative care. During the hospital stay, the palliative care team was involved, and a family meeting with the entire care team was convened to discuss the future plan of care. The decision, made by the family members and the patient's power of attorney, was to transition to comfort care measures. Although the patient did not have any advance care planning documented on file, discussions with the family clarified and confirmed this decision. Throughout the hospital stay, the patient’s mental status and overall condition deteriorated. As a result, the patient was transferred to the local Veteran Affairs hospital for general inpatient hospice care. The patient continued to experience a rapid, progressive decline and passed away during their time in inpatient hospice care within the following weeks.

## Discussion

PML is a rare demyelinating disorder caused by the reactivation of the JCV, leading to neurologic impairment and poor prognosis. The majority of PML cases occur in the following populations: 80% in HIV-positive individuals, 10% in those with hematologic malignancies, and less than 5% in patients with relapsing-remitting multiple sclerosis treated with natalizumab [9]. PML rarely occurs in MM. Upon reviewing previous cases describing PML in MM, 13 out of 17 reported prior treatment regimens that included IMiDs [7,8]. Considering the concurrence of PML in MM patients who are on IMiDs, our case provides further suggestion of an association between IMiD use and increased risk of PML.

The mechanism of IMiDs causing a higher incidence of PML in MM patients is unclear, but we present a possible link based on existing knowledge of the interlinking pathophysiology of PML and MM and the pharmacology of IMiDs. In PML, viral DNA is reactivated in both CD34+ stem cells and B lymphocytes. Infected lymphocytes then traffic the JCV to the brain [10]. T-cell dysfunction also plays a role in the immunocompromised state required for JCV reactivation. The higher incidence of PML in patients with HIV/AIDS and idiopathic CD4+ T-cell lymphopenia underscores the importance of CD4+ T lymphocytes in the pathophysiology of PML. Low CD4+ and CD8+ T-cell counts or evidence of their impaired function have been associated with a large majority of PML cases [11].

Multiple myeloma (MM) can also lead to T-cell dysfunction, as Witzens-Harig et al. demonstrated that MM cells express carcinoembryonic antigen-related cell adhesion molecules (CEACAMs) that bind to and crosslink with CD8+ T cells, resulting in the inhibition of T-cell activation [12]. There is also evidence of a decreased ratio of CD4+ to CD8+ T cells, along with an association of higher CD8+ HL-DR+ cells in patients with a poorer prognosis [13]. This T-cell dysregulation, along with reduced CD4+ T-cell function and altered CD8+ T-cell activity, can create a permissive environment for viral reactivation, contributing to JCV reactivation in the setting of PML. Furthermore, MM may also lead to impaired B-cell function, disrupting the normal immune surveillance mechanisms, and enhancing the vulnerability to JCV infection. It can then be concluded that the T-cell dysregulation in MM plays a role, alongside IMiDs, in creating a background of immunodeficiency for PML to take place.

The mechanism of action of IMiDs is complex and not fully understood but includes downregulating nuclear factor kappa-light-chain-enhancer of activated B cells (NF-κB) signaling, inducing Jun N-terminal kinase (JNK)-dependent caspase-8-mediated apoptosis, decreasing adhesion molecules like intercellular adhesion molecule 1 (ICAM-1) and vascular cell adhesion molecule 1 (VCAM-1), inhibiting interleukin-6 (IL-6) and vascular endothelial growth factor (VEGF), and disrupting insulin-like growth factor 1 (IGF-1) and fibroblast growth factor (FGF) genes to prevent angiogenesis. Together, these actions disrupt tumor growth and proliferation in MM [14]. Lenalidomide and pomalidomide, developed after thalidomide, have also been found to induce the proliferation of CD4+ and CD8+ T cells while inhibiting regulatory T cells (Tregs) [15]. Despite this apparent immune-stimulatory role of IMiDs, they can also affect immune cell dynamics in more complex ways, particularly regarding B-cell function. IMiDs can suppress malignant B-cell proliferation, which may inadvertently affect the overall pool of B lymphocytes, including those tasked with mounting a defense against viral infections like JCV. This suppression of B cells, in combination with altered T-cell function, can further impair immune responses and increase susceptibility to PML. This raises a possible conflictive narrative, as IMiDs would appear to be protective against PML as opposed to a predisposing factor. However, IMiDs have been shown to have an enhancing effect with rituximab, an anti-CD20 monoclonal antibody, in destroying malignant B-cells and decreasing peripheral B cell counts [16]. Rituximab use in a variety of patient populations has been shown in observational studies to be associated with a higher risk of PML [17,18]. The pathophysiology of rituximab-associated PML remains unclear with the thought that after B-cell differentiation, pre-B cells with latent JCV are released into the circulation. The humoral immune response may play a larger role in defending against JCV than initially thought as there are cases of PML in those with congenital disorders of humoral immunity [17]. With the full pharmacological impact of IMiDs having yet to be uncovered, it can be postulated that, similar to rituximab, their effects on B cells may increase the risk of PML.

## Conclusions

PML associated with MM is a rare complication and is associated with high mortality. This case contributes to the few existing reports and supports an association between the use of IMiDs in MM and increased risk of development of PML. With many patients receiving immunomodulator therapies, it is important to closely monitor neurological changes in these patients with PML on the differential. With our patient’s presentation of neurological abnormalities and a subsequent diagnosis of PML while on an IMiD, our case serves as supporting evidence for PML as a potential complication of IMiD use. Such awareness can better prepare physicians to have early recognition of new-onset neurocognitive symptoms suggestive of PML in patients undergoing IMiD therapies, allowing for timely intervention. Further research is suggested to invest in surveillance programs for JCV infection after initiating IMiD therapy.
